# A New Mouse Model for Mania Shares Genetic Correlates with Human Bipolar Disorder

**DOI:** 10.1371/journal.pone.0038128

**Published:** 2012-06-04

**Authors:** Michael C. Saul, Griffin M. Gessay, Stephen C. Gammie

**Affiliations:** 1 Department of Zoology, University of Wisconsin–Madison, Madison, Wisconsin, United States of America; 2 Neuroscience Training Program, University of Wisconsin–Madison, Madison, Wisconsin, United States of America; University of Iowa Hospitals & Clinics, United States of America

## Abstract

Bipolar disorder (BPD) is a debilitating heritable psychiatric disorder. Contemporary rodent models for the manic pole of BPD have primarily utilized either single locus transgenics or treatment with psychostimulants. Our lab recently characterized a mouse strain termed Madison (MSN) that naturally displays a manic phenotype, exhibiting elevated locomotor activity, increased sexual behavior, and higher forced swimming relative to control strains. Lithium chloride and olanzapine treatments attenuate this phenotype. In this study, we replicated our locomotor activity experiment, showing that MSN mice display generationally-stable mania relative to their outbred ancestral strain, hsd:ICR (ICR). We then performed a gene expression microarray experiment to compare hippocampus of MSN and ICR mice. We found dysregulation of multiple transcripts whose human orthologs are associated with BPD and other psychiatric disorders including schizophrenia and ADHD, including: Epor, Smarca4, Cmklr1, Cat, Tac1, Npsr1, Fhit, and P2rx7. RT-qPCR confirmed dysregulation for all of seven transcripts tested. Using a novel genome enrichment algorithm, we found enrichment in genome regions homologous to human loci implicated in BPD in replicated linkage studies including homologs of human cytobands 1p36, 3p14, 3q29, 6p21–22, 12q24, 16q24, and 17q25. Using a functional network analysis, we found dysregulation of a gene system related to chromatin packaging, a result convergent with recent human findings on BPD. Our findings suggest that MSN mice represent a polygenic model for the manic pole of BPD showing much of the genetic systems complexity of the corresponding human disorder. Further, the high degree of convergence between our findings and the human literature on BPD brings up novel questions about evolution by analogy in mammalian genomes.

## Introduction

Bipolar disorder (BPD) is a psychiatric disorder characterized by episodic mania and depression [1]. It is a common mental health problem, with an estimated lifetime prevalence of approximately 1–5% [2], [3]. A meta-analysis of family, twin, and adoption studies found that relatives of BPD patients have a 10-fold higher risk of the disorder than those without relatives with BPD [4], demonstrating that BPD has a strong heritable constituent. Though ongoing efforts to elucidate the genetic basis of BPD using varied approaches have yielded promising results, a convincing molecular etiology of BPD remains elusive [5]. There are at least a few good reasons for this difficulty in finding a genetic basis for BPD. First, BPD is a complex disorder at the molecular level, involving perturbations of not just single genes, but of systems of genes [6]. Second, it may be more proper to speak of bipolar disorders in the plural; the pathology may have multiple heterogeneous molecular bases [6], [7], a hypothesis consistent with the multiple heterogeneous findings in different genome-wide studies of BPD [8]. Third, deriving mechanistic explanations of human psychiatric disorders using classical genetics presents difficulties due to practical constraints on experimental power and the possibility of epigenetic components of these disorders [5], [9]–[11].

Because a convincing BPD molecular etiology poses significant technical and theoretical challenges to human geneticists, animal models for BPD have a strong potential to extend understanding of this disorder. The main animal modeling approach to date has been the use of separate rodent models for mania and depression [12]. Models for the manic pole of BPD have primarily utilized treatment with psychostimulants [13], [14] or single locus transgenic approaches like dopamine transporter knockouts [15], [16] and various molecular clock gene knockouts [17], [18], though the Black Swiss strain has recently been proposed as a tentative naturally-occurring mania model [19], [20]. Importantly, there exists at least one strain of rodent, the Flinders Sensitive Line (FSL) of rat, which shows a well-validated depressed phenotype relative to control strains [21]. This strain of rat has been used in multiple studies to examine several molecular aspects of depression [22]–[24], and it has been useful in conceptualizing depression as a disorder with a complex molecular etiology [12], [25].

Our lab recently characterized a tentative model for the manic pole of BPD [26]. This model, an inbred mouse strain termed Madison (MSN), displays a naturally manic phenotype. Relative to control strains, MSN mice show increased locomotor activity, increased forced swimming, decreased sleeping, and increased sexual activity. Further, treatments with both lithium chloride and olanzapine moderate the MSN manic phenotype, a necessary condition for a predictively valid model for the manic pole of BPD [27].

Our initial behavioral and pharmacological characterization of the MSN strain showed promise, but without molecular correlates, the model lacked construct validity. Consequently, we performed a gene expression microarray study with RT-qPCR confirmation to extend the phenotype of the MSN mouse relative to their ancestral outbred hsd:ICR (ICR) strain. When choosing which brain region to interrogate, we decided to look at gene expression in hippocampus. In humans, hippocampus shows microstructural and functional differences in BPD patients as assayed by MRI [28], [29]. Further, post-mortem analyses of hippocampal tissue from BPD patients show multiple histological and gene expression differences relative to hippocampus from psychiatrically normal controls [29]–[32]. Prior to the microarray experiment, we decided to replicate the most robust measure from our previous behavioral work, total locomotor activity, to confirm that MSN mice stably display a manic phenotype.

## Results

### Total Locomotor Activity

**Figure 1:**
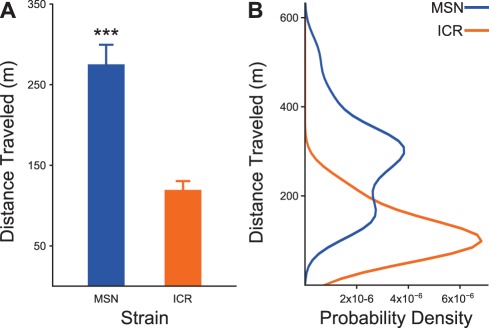
Confirmation of the MSN manic phenotype using an experimental replication of the most robust behavioral measure from previous research on this mouse strain, total locomotor activity. A) MSN mice display stable heightened locomotor activity relative the outbred strain. ***P<0.001. B) The probability density for MSN mouse total locomotor activity is bimodal, while the probability density for the control strain is unimodal. This leads us to the hypothesis that MSN mice may display behavioral bipolarism, a hypothesis that will be examined in future work.

Locomotor activity defined by total distance traveled (Fig. 1.A) was significantly higher in MSN mice than in ICR mice (P-value = 7×10^−7^, Monte Carlo permutation test, n_MSN_ = n_ICR_ = 19, Z = −4.24, B = 1×10^7^). The probability density distribution for total distance travelled for MSN mice was bimodal whereas the probability density distribution for ICR mice was unimodal (Fig. 1.B). Since the MSN strain is almost completely inbred, we do not believe this bimodality is evidence of two separate populations within the MSN strain.

### Single Gene Microarray Results

**Figure 2:**
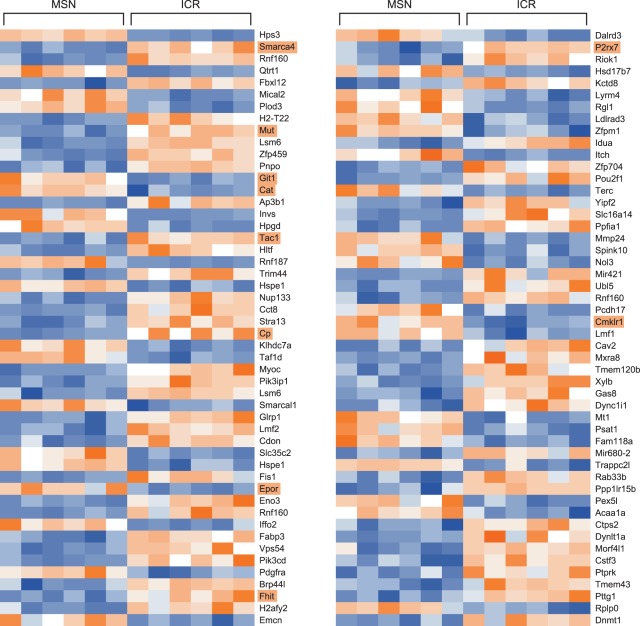
Heatmap of normalized values for the top 100 well-annotated genes from the microarray experiment listed by P-value in order from top to bottom, then left to right. The lowest P-values are at the top left corner, and the highest are at the bottom right. Blue values represent lower expression and orange values represent higher expression. The names of the genes discussed in the text of this manuscript are highlighted in light orange.

The hundred best annotated genes with the most significant P-values along with a heatmap showing expression in each of the tested samples are listed in Fig. 2. MSN mice showed significant differences in gene expression in multiple genes whose orthologs are associated with BPD and the related mental health disorders schizophrenia, depression, and ADHD in the human literature. Significantly-dysregulated genes (P-value <1×10^−3^) whose human orthologs have been associated with these disorders in at least two separate studies include: Cp (P-value = 1.25×10^−5^, t = −7.421) [33]–[35], Epor (P-value = 2.73×10^−5^, t = 6.823) [36]–[38], Pdgfra (P-value = 4.38×10^−5^, t = 6.476) [39], [40], Tac1 (P-value = 9.03×10^−6^, t = −7.684) [41]–[43], P2r×7 (P-value = 6.72×10^−5^, t = −6.170) [44]–[49], Fhit (P-value = 5.20×10^−5^, t = −6.352) [50], [51], and Cat (P-value = 6.56×10^−6^, t = 7.946) [52], [53]. Significantly-dysregulated genes whose human orthologs have been associated with BPD or a related mental health disorder in one study include: Smarca4 (P-value = 7.58×10^−7^, t = −9.897) [54], Mut (P-value = 3.44×10^−6^, t = −8.495) [55], Git1 (P-value = 6.24×10^−6^, t = 7.988) [56], and Cmklr1 (P-value = 1.12×10^−4^, t = 5.818) [57]. An additional dysregulated gene of interest we identified whose human ortholog has not been associated with BPD or related mental health disorders as far as we know is Npsr1 (P-value = 9.48×10^−4^, t = 4.455). This gene’s product is a G-protein coupled receptor generally involved with arousal and activity [58], [59]. The names of genes discussed here are highlighted in light orange in Fig. 2. All reported values use an empirical Bayesian t-test with 10 degrees of freedom.

### RT-qPCR Confirmation of Microarray Results

**Figure 3:**
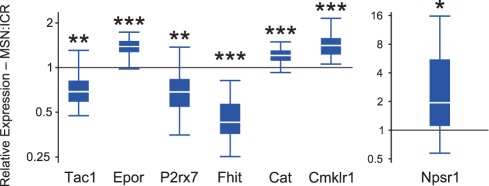
RT-qPCR confirmation results for seven genes from the microarray. Ratio distribution is graphed as a box-and-whiskers plot. Ratios greater than 1 represent genes with higher expression in the MSN strain and ratios less than 1 represent genes with lower expression in the MSN strain relative to the ICR strain. *P<0.05; **P<0.01; ***P<0.001.

We confirmed the results of seven genes from our microarray experiments using RT-qPCR. We chose genes for confirmation with an emphasis on gene products that we thought were either related to neural signaling pathways it would be possible to target pharmacologically or genes we could use as dependent variables in the future. We tested Cat (P-value = 0.001, expression ratio = 1.202), Cmklr1 (P-value<0.001, expression ratio = 1.404), Epor (P-value<0.001, expression ratio = 1.370), Fhit (P-value<0.001, expression ratio = 0.446), Npsr1 (P-value = 0.021, expression ratio = 2.426), P2rx7 (P-value = 0.006, expression ratio = 0.681), and Tac1 (P-value = 0.001, expression ratio = 0.696). The results of the RT-qPCR confirmation are shown in Fig. 3. All seven genes we chose to confirm were found significantly dysregulated in the same direction and at the same approximate magnitude as in the results of the microarray experiment. Altogether, our RT-qPCR confirmation provides evidence that our microarray data are fundamentally sound.

### Genome Enrichment Analysis

**Figure 4:**
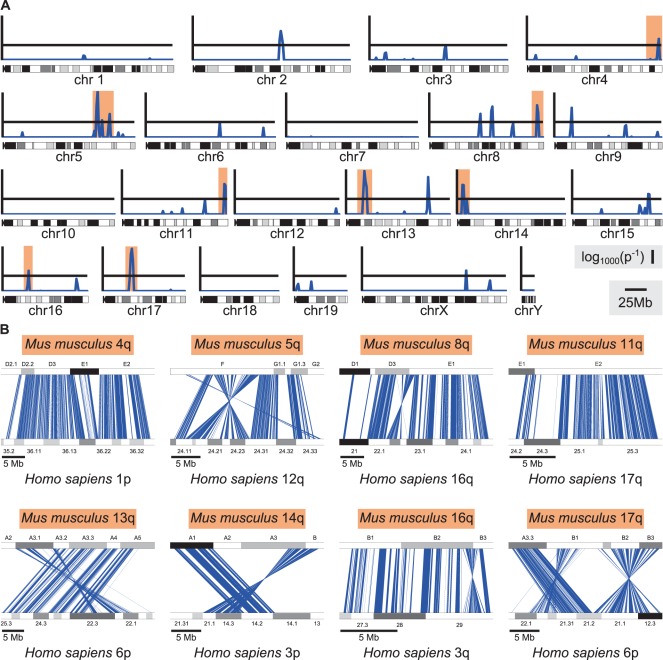
Genome enrichment analysis and homology of highlighted enriched clusters to the human genome. A) Genome enrichment analysis of the MSN phenotype using a novel enrichment algorithm we created for this study (see Materials and Methods). The y-axis represents the log_10_ inverse of the corrected binomial probability that a cluster of dysregulated genes would occur by chance. The black horizontal lines demarcate a cluster occurring by chance with a 0.001 corrected probability, consistent with a LOD or NPL score of 3 in a linkage study. Spikes above the black lines indicate dysregulated gene clusters highly unlikely to occur by chance, indicating that the genome region is significantly enriched. Corrected probabilities less than 1×10^−9^ are collapsed to 1×10^−9^. B) Shared synteny, a similar clustering of orthologous genes, between the clusters on the murine genome highlighted in orange in Fig. 4.A and human genome regions strongly implicated in BPD (see Results for details). Blue lines represent orthologous genes and their positions in the murine (upper) and human (lower) genomes.

NIAID DAVID functional annotation analysis by cytoband of all genes dysregulated at P<0.01 found a significant enrichment in murine cytoband 5qF (3.789-fold enrichment, Bonferroni-corrected P-value = 7.84×10^−7^) in MSN mice. We found this result intriguing, but we believed this cytoband-style enrichment analysis utilized genome regions too wide to allow the assumption of classical genetic linkage. We created a new algorithm for genome enrichment analysis with much narrower partitions of the genome queried. Our novel genome enrichment analysis yields results that look very similar to a conventional genome-wide linkage or association study, and we find it useful for generating predictions for broad chromosomal regions potentially related to a given population’s phenotype. We found significant enrichment in a total of fifteen genome regions (Fig. 4.A).

When looking at the significantly enriched genome regions, we decided to examine their relationship to the human genome, so we qualitatively looked at shared synteny, a similar clustering of orthologous genes between species that generally demarcates genome homology. We looked for linkage and association literature implicating the enriched regions’ human homologs in BPD and related mental health disorders like schizophrenia and ADHD. We classified a human genome region implicated in BPD in at least two linkage studies with at least one study showing a LOD or NPL score (linkage score) greater than 3 as a region with a strong relationship to BPD. Using this criterion, we found that eight of the enriched genome regions in MSN mice are homologous to seven human genome regions displaying strong relationships to BPD. Shared synteny for these regions is shown in Fig. 4.B. These regions include the following cytobands: murine 4qE, homologous to human 1p36, implicated in BPD in two studies with linkage scores of 3.97 [60] and 3.1 [61] and a region in which SNPs predict BPD susceptibility [62]; murine 5qF, homologous to human 12q24, implicated in BPD in multiple studies with linkage scores of 4.91 [63], 3.63 [64], 3.37 [65], 2.8 [61], and 2.08 [66] and a region in which SNPs and allele variants predict BPD susceptibility [44], [46], [67]; 8qE1, homologous to human 16q24, implicated in BPD in two studies with linkage scores of 3.51 [68] and 2.29 [69]; murine 11qE2, homologous to human 17q25, implicated in BPD in five studies with linkage scores of 3.11 [70], 2.4 [71], 2.4 [72], 2.1 [73], and 2.08 [74]; murine 13qA3 and 17qA3-17qB1, two cytobands with homology to human 6p21–22, implicated in BPD in multiple studies with linkage scores of 3.19 [68], 2.60 [75], 2.26 [72], and 1.91 [69]; murine 14qA1, homologous to human 3p14, implicated in BPD in two studies with linkage scores of 3.51 [76] and 2.31 [77]; and murine 16qB2–B3, homologous to human 3q29, implicated in BPD in two studies with linkage scores of 3.74 [78] and 2.0 [61]. Additional enriched genome regions showing weaker previous relationships to BPD included: murine 2qE, homologous to human 11p13, implicated in BPD in one study with a linkage score of 1.95 [79] and a region in which SNPs and allele variants predict BPD susceptibility [80], [81]; murine 8qB2–B3.1, homologous to human 4q34, implicated in BPD in a study with a linkage score of 3.28 [82]; murine 8qB3.3 and 9qA3, homologous to human 19p13, implicated in BPD in three studies with linkage scores of 2.37 [83], 1.8 [66], and 1.55 [84]; and 15qE3, homologous to 22q13, implicated in BPD in one study with a linkage score of 2.22 [85]. One cluster at murine cytoband 13qD1 shared synteny with human 5q13–14, a region with no significant linkage to BPD in any human literature we could find, but implicated in schizophrenia in a study with a linkage score of 3.20 [86] and implicated in ADHD in a study with a linkage score of 4.16 [87]. A cluster on the X chromosome consisted of mostly predicted genes with no obvious orthology to known human genes. We were not able to find shared synteny between this region and any human region.

### Functional Network Analysis

**Figure 5:**
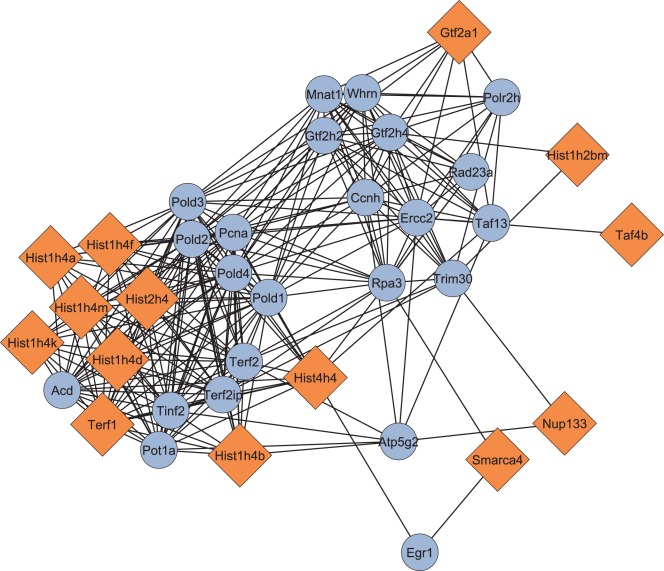
Network analysis of a dysregulated gene network related generally to chromatin packaging. The diamond-shaped orange nodes indicate genes found significantly dysregulated MSN mice while the circular blue nodes indicate related genes called by the MiMI plugin for Cytoscape. A recent systems meta-analysis of human BPD genome and transcriptome studies found that a significant chromatin packaging effect is seen across multiple human BPD populations [54].

NIAID DAVID analysis of all genes with FDR-adjusted P-values less than 0.25 found a significant gene cluster generally related to chromatin packaging, so we decided to pursue this finding using Cytoscape to visualize this cluster. Using a gene list generated from this DAVID analysis, we found a network of genes generally related to chromatin packaging that was significantly dysregulated in our microarray study. These genes included a few histone-related genes as well as Smarca4, a gene we found to be one of the most highly-dysregulated in the microarray results that has helicase and chromatin remodeling activities. The chromatin packaging gene network is shown in Fig. 5, with nodes in orange representing those genes found dysregulated in our microarray results and nodes in blue representing linking genes previously found related to chromatin packaging not significantly dysregulated in our microarray. These results suggest that differential chromatin packaging is part of the MSN phenotype, which is convergent with the findings of a recent systems biology meta-analysis of BPD [54].

## Discussion

### A Complex Phenotype

As BPD is a genetic disorder involving systems of genes [6], [8], [54], models for either pole of BPD representing single genes, while valuable for many purposes, are necessarily limited. A mammalian genome containing upwards of 30,000 genes is a complex system, and while understanding the effects of single genes and their products will always be necessary, their results should be understood in a systems biology context. We believe that the MSN strain represents among the first true systems biology models for the manic pole of BPD characterized. The polygenic nature of this model presents both challenges and promise. The quantity of loci involved in the phenotype makes inference difficult relative to single locus models, but it reproduces the physiology of the disorder more completely and more subtly. We believe the MSN mouse strain will enable us to glean new insights into the biology of BPD based upon not only face validity, but on fundamental biological construct validity.

The basis of this polygenic phenotype is the MSN strain’s ∼15 year history of multifaceted inbreeding. The ancestral strain of these mice was one of four replicate strains originally bred for high wheel running in a study on exercise physiology [88]. The ancestors showed few notable exercise physiological changes; the most significant changes observed in this strain displayed were neurological [89]. These MSN ancestors were part of a 2003 hippocampus microarray paper comparing selectively bred strains with control strains [90]. None of the 53 genes found significantly dysregulated in the 2003 microarray experiment overlap with the significantly dysregulated genes from the present study, and most of these 53 genes are quite far from significance in the current array. While methodological differences prevent easy comparisons between the 2003 microarray and the current microarray, we believe the fundamental divergence of the two arrays implies that the chief physiological changes we see in the MSN strain likely emerged after the MSN progenitors were selectively-bred for high wheel running. The sole selection event occurring after the original microarray experiment was ∼30 generations of selection to maintain a trait already observed in the MSN progenitors, high maternal defense [91]. After this maintenance selection, the MSN strain’s ancestors were maintained in our lab without selective breeding. Given the lack of breeding toward a novel phenotype, we believe many of the important genetic changes contributing to the MSN phenotype are likely attributable to genetic drift. This is not to deemphasize the effect of the original selective breeding for high wheel running, which provided the genetic foundation of the MSN strain. We suggest that the MSN strain’s manic phenotype emerged through a series of random events acting upon an already constrained gene pool.

The MSN mouse strain’s locomotor activity displays a bimodal distribution while the outbred mouse strain’s locomotor activity shows a unimodal distribution. Because the MSN strain is highly inbred, we believe this bimodality does not suggest divergent populations of mice within the MSN strain. Instead, we speculate that the MSN strain may show a phenotype with true behavioral cyclicity, a finding that has never been seen in a rodent model as far as we know. We are currently working on a behavioral project to examine this possibility in greater detail.

### Implications of the Genome Findings

The genome enrichment algorithm we developed is a new and useful method extending the suite of systems biology tools for high-throughput gene expression data. This algorithm can be applied to gene expression datasets old and new to enhance the biological understanding of the genome as a substrate for the organization of gene systems. Importantly, we believe it predicts both potential perturbations of the genome and of the epigenome, which would provide a more complete accounting of the mechanistic underpinnings of differences on the genome than classical genetics can. When used in concert with classical genetic techniques, we believe this technique has the potential to inform biologists not only about where to look for differences on the genome, but for which types of differences they should interrogate each genome regions.

While this genome enrichment analysis does not substitute for a true genome-wide linkage scan, we believe it provides a strong prediction that the MSN genome experiences perturbations in areas homologous to human genome regions linked to BPD in some populations. Why human populations with BPD should show differences in the genome relative to psychiatrically normal comparisons is an interesting question. Why MSN mice might share some of the same genomic perturbations as some human BPD populations is a compelling extension of this question. We propose that because the structural and functional components of mammalian genomes do not differ significantly, these genomes, given analogous evolutionary events, display analogous changes. Put more simply, similar genomes experiencing similar forces react similarly. In this case, we believe we may have found a conserved genomic signature observable even after the some 75 million years since the divergence of the mouse and human lineages [92]. We speculate that this signature is related generally to neural activation and organismal arousal. Further, our findings suggest that conserved genomic signatures may exist for other disorders and traits.

### Conclusions

Though these results show promise, we must include caveats based upon the complex nature of the phenotype and the limitations of the techniques we used. MSN mice weigh significantly less than ICR controls (t_27.957_ = −3.986, P-value = 4.369×10^−4^, Welch’s two-sample t-test), which shows that the MSN background includes other characteristics potentially unrelated to mania. While the microarray platform we used was designed to be robust against the effects of polymorphisms in the probes, ultimately, we cannot preclude the possibility of coding changes in the probe binding sites of some genes of interest affecting our results. Similarly, while we made all practical efforts to design qPCR oligonucleotides on monomorphic sites, we cannot say with certainty that our primer binding sites do not contain novel polymorphisms. Additionally, as we noted in the results, some of the evidence from the human literature we utilized to contextualize our results is unreplicated.

Despite these complexities, when we look at the rich suite of systems biology differences present in MSN strain relative to the closely related ICR strain, we believe we have found a strong phenotype that models mania with high construct validity. We have demonstrated that MSN mice reiterate a substantial amount of work done on human BPD genetics using three levels of analysis. At the single gene level, MSN mice display dysregulation of multiple transcripts whose human orthologs are related to BPD and related mental health disorders [8], [33]–[57]. At the systems level, MSN mice display dysregulation of a gene network similar to one found conserved across multiple human studies on BPD in a recent systems meta-analysis [54]. At the chromosomal level, MSN mice display perturbations in eight murine genome regions homologous to seven human genome regions with strong relationships to BPD in the human literature [60]–[78]. We believe that the argument for analogy between MSN mice and human BPD is strong. The genetic, systems, and genome findings we present here imply profound physiological similarities.

Just as the etiology of human BPD remains unresolved [5], so do the mechanistic underpinnings of the MSN strain’s phenotype. This study is an extension of our effort to characterize the phenotype and a preliminary step in the process of finding a genotype. While we believe we have fully utilized a strong dataset to glean an interesting picture of these mice, until we understand more about the genomics behind the MSN phenotype, the scope of our work remains limited. We believe the loci from our novel genome enrichment analysis give us a set of targets relevant to a potential deep sequencing project. Comparing the MSN genome with the outbred ICR genome will be an important next step and will contribute much to our understanding of BPD.

## Materials and Methods

### Ethics Statement

Animal use was carried out in accordance with the recommendations in the Guide for the Care and Use of Laboratory Animals of the National Institutes of Health. All protocols were approved by the University of Wisconsin–Madison IACUC (protocol #: L00405-0-05-09), and all reasonable efforts were made to minimize animal suffering.

### Animals

MSN is an inbred strain of mouse derived over the course of approximately 15 years from the outbred hsd:ICR (ICR) mouse strain (Harlan Laboratories, Madison, WI, USA), making the ICR strain a natural control. The ancestors of MSN mice were one of four replicate strains selected over a period of ∼30 generations for high wheel running behavior [88]. This high wheel running ancestral strain was observed to display high maternal defense behavior compared to both control lines and the other three lines selected for high wheel running from the original selective breeding experiment [91]. The ancestral mice were then bred for an additional ∼30 generations to maintain high maternal defense behavior. This progenitor strain was also characterized as showing maternal neglect [93]. The mice were maintained in a breeding colony in our lab without selection for multiple generations, likely experiencing genetic drift and fixation before our lab eventually observed them to display a manic phenotype relative to control strains [26]. The MSN strain is now highly inbred; we estimate its current inbreeding coefficient at 0.95. The MSN and ICR strains were kept in separate breeding colonies in our laboratory under similar conditions for multiple generations prior to this study. Mice from this study were adult males from the same generation singly housed in the same room, and all mice were approximately 10 weeks old during testing.

### In-cage Locomotor Activity Observations

Total in-cage locomotor activity observations during portions of both the light and dark periods were made using a camera mounted above mouse home cages with online analysis done by the TopScan 2.0 software (CleverSys, Reston, VA, USA) as described previously [26], though for this experiment, we observed MSN mice during one day and not two. In total, 19 MSN mice and 19 ICR mice were observed. Statistical inference for total distance traveled over time tested was done using the Monte Carlo permutation test implementation in the R package coin. Probability density plots were made using the R package sm, a non-parametric smoothing algorithm for histograms.

### Tissue Collection

The day after observation of total locomotor activity, mice were weighed, anesthetized with isofluorane gas, and decapitated. After whole brains were removed, hippocampal tissue was quickly dissected out and the hippocampi were flash-frozen over dry ice. Hippocampal tissue was kept frozen at −80°C for no longer than 12 weeks prior to use in downstream molecular biology applications. Statistical inference on animal weight was performed using the t.test function in R.

### RNA Extraction

Total RNA was extracted using an Aurum Total RNA Fatty and Fibrous Tissue Kit (Bio-Rad, Hercules, CA, USA) to manufacturer specifications. Briefly, tissue was disrupted in a low pH phenol-chloroform-guanidium thiocyanate solution and spun down. The aqueous phase was combined with ethanol and passed through a spin column to bind RNA. The RNA was cleaned and DNase treated on-column, purified total RNA was eluted in nuclease-free elution buffer, and samples were frozen at −80°C prior to use in downstream applications.

### Microarray Target Preparation, Hybridization, and Scanning

Using the true random number generation service random.org, 6 mice from the MSN group and 6 mice from the ICR group were chosen at random from the mice used in behavioral observations. Prior to the microarray experiment, all total RNA samples were checked for purity, integrity, and concentration using a NanoDrop ND-1000 spectrophotometer (Thermo Fisher, Waltham, MA, USA) in concert with RNA 6000 Pico Chips in a 2100 BioAnalyzer (Agilent Technologies, Santa Clara, CA, USA). The microarray experiment utilized the GeneChip Mouse Gene 1.0 ST platform (Affymetrix, Santa Clara, CA, USA), a platform with probesets consisting approximately 27 probes spaced along the length of each gene interrogated to minimize the effects of polymorphic sites on the expression results for the gene as a whole, with biotinylated targets derived from total RNA. Briefly, cDNA for hybridization was synthesized from 400 ng of total RNA using a GeneChip WT Expression Kit (Ambion, Austin, TX, USA) according to the manufacturer’s specifications. The cDNA was fragmented, then biotinylated using a WT Terminal Labeling Kit (Affymetrix, Santa Clara, CA, USA) according to the manufacturer’s specifications. Biotin-labeled cDNA was hybridized with microarrays at 45°C for 16 hours. The hybridized arrays were washed, stained, and scanned on a GC3000 G7 Scanner (Affymetrix, Austin, TX, USA). Data were extracted and processed from the scanner using the Affymetrix Command Console software, v. 3.1.1.1229. Microarray target preparation, hybridization, and scanning were performed by the Gene Expression Center, a microarray core lab at the University of Wisconsin–Madison.

### Probeset Level Normalization, Summarization, and Statistical Inference

Probeset-level normalization and summarization were performed with the PLIER algorithm with GC-bin background correction using the Affymetrix Power Tools software, v. 1.12.0 and revision 4 of the Affymetrix library for the Mouse Gene 1.0 ST version 1 array platform. The raw and summarized microarray data discussed in this publication have been deposited in the NCBI’s Gene Expression Omnibus [94] and are available through the GEO website, accession number GSE29417. Inferential statistics for differential expression between MSN and ICR samples were calculated using the microarray-specific empirical Bayesian t-test implementation in the Bioconductor package limma, v. 3.6.9 [95], to calculate nominal P-values and Microsoft Excel 2010 to calculate linear fold-change differences. A tab-delimited spreadsheet containing exhaustive microarray statistical results with annotations from both Affymetrix, a reliable annotation source, and fill-ins for transcript clusters unannotated by Affymetrix from Ensembl’s BioMart, a less reliable annotation source, is posted as supplementary Table S1.

### RT-qPCR Validation of Microarray Results

We have included an RT-qPCR supplement, Table S2, which contains all of the information about the RT-qPCR methods from this study required by the MIQE [96].

A total of 8 MSN mice and 8 ICR mice were selected for RT-qPCR confirmation. An additional 2 MSN mice and 2 ICR mice were selected using random.org from the same set of mice for RT-qPCR confirmation to add to the 6 MSN and 6 ICR mice used in the microarray experiment. RNA extraction from hippocampi of the additional animals was done at the same time as extraction for the animals used in the microarray experiment. Fresh aliquots of RNA were used for RT-qPCR confirmation to avoid samples differentially exposed to freeze-thaw cycles. Prior to RT-qPCR confirmation, total RNA was checked for purity, integrity, and concentration using both a NanoDrop ND-1000 spectrophotometer and RNA 6000 Nano Chips in an Agilent 2100 BioAnalyzer. Results of quality control are reported in table S2.10.

Seven gene transcripts found significantly dysregulated in the microarray were chosen for validation by RT-qPCR: catalase (Cat); chemokine-like receptor 1 (Cmklr1); erythropoietin receptor (Epor); fragile histidine triad (Fhit); neuropeptide S receptor 1 (Npsr1); purinergic 2× receptor 7 (P2rx7); and tachykinin, precursor 1 (Tac1). As they were recently demonstrated among the most stable gene transcripts of the widely used RT-qPCR reference genes in the rodent brain [97], [98], the transcripts for tyrosine 3-monooxygenase/tryptophan 5-monooxygenase activation protein, zeta polypeptide (Ywhaz) and succinate dehydrogenase complex, subunit A, flavoprotein (Sdha) were chosen as reference genes for this study. Primers for all transcripts were designed in NCBI’s online Primer-BLAST software suite set to use strict *in silico* specificity requirements and to preclude primer binding sites on known polymorphic sites. Primer sequences are reported in table S2.11.

We used a two-step protocol for RT-qPCR. Reverse transcription was done using a SuperScript III First-Strand Synthesis System for RT-PCR (Invitrogen, Carlsbad, CA, USA) according to the manufacturer’s specifications. 2 µg of total RNA were used as template for each sample, a poly-T 20 mer priming strategy was used, and reaction volumes were scaled up to 26.25 µL. Reverse transcription reactions were done in a MasterCycler Personal PCR Machine (Eppendorf, Hamburg, Germany). The resultant cDNA was diluted 1∶5 with nuclease-free water to minimize the effects of any PCR inhibitors. The specifics of cDNA synthesis are listed in table S2.4.

Real-time quantitative PCR was done in a StepOnePlus real-time thermal cycler (Applied Biosystems, Foster City, CA, USA) using SsoFast EvaGreen Supermix (Bio-Rad, Hercules, CA, USA) according to manufacturer recommendations. Total reaction volumes of 20 µL were used; each reaction contained 10 µL of 2× Supermix, 2 µL diluted template cDNA, 500 nM forward and reverse primers, and nuclease-free water up to the final reaction volume. All reactions were performed in triplicate. We utilized a three-step thermal cycling protocol that included a 30 s hot start at 95°C, then 40 cycles of a denaturation step at 95°C for 5 s, an annealing step at a temperature empirically-determined for each primer set for 20 s, and an elongation step at 72°C for 20 s. Annealing temperatures for each primer set are available in table S2.4. Fluorescence data were collected at the annealing step of each cycle. All experimental qPCR runs were accompanied by a dilution series to calibrate PCRs for empirical efficiency and by a dissociation curve to determine *in vitro* primer specificity. All consumables are listed in table S2.7.

C_q_ values were determined using the StepOnePlus software, v. 2.1, with the same fluorescence threshold for every qPCR run. Descriptive and inferential statistics were calculated using the Relative Expression Software Tool (REST), v. 2009, which corrects for empirical PCR efficiency, allows for the use of multiple reference genes, and utilizes a Monte Carlo style permutation test for significance [99]. We set the REST software to perform 10,000 iterations for the permutation test, and we also used it to find expression ratio of MSN:ICR.

### Genome Enrichment Analysis and Shared Synteny Analysis

To map genome regions with significant enrichment, we built a novel enrichment algorithm that looks at clustering of dysregulated genes along the length of a chromosome. This algorithm works by walking along the length of a chromosome at 1.25 Mb intervals, a distance corresponding to a little less than 2.5 centimorgans in mice. The algorithm bins all genes assayed in our microarray platform within 1.25 Mb of the center of each interval and counts them. The bins are staggered to prevent bias against clustering at bin breakpoints. The algorithm counts all genes dysregulated at a nominal P-value of 0.01 or less. The amount of genes significantly dysregulated within each interval should, under a null hypothesis of no significant enrichment of that genome region, display a binomial distribution with a probability of any given gene being significantly dysregulated at no more than 0.01. Our algorithm calculates the binomial probability of the amount of the amount of dysregulated genes within each interval over the entire genome. The probabilities are Bonferroni-corrected for multiple comparisons, then the log_10_ of the inverse of the probabilities are graphed. A log_10_(p^−1^) of 3 indicates a Bonferroni-corrected probability of a cluster occurring 1 in 1,000 times under the null hypothesis of no significant clustering in any particular genome region. This corresponds to the LOD score of 3 commonly used as the cutoff criterion for strong evidence of linkage in linkage scans. Any log_10_(p^−1^) value greater than or equal to 9 is collapsed to 9 for ease of visualization. The resulting graphic examines clusters over the whole genome, and spikes indicate clusters highly unlikely to have occurred by chance. The Excel file used to create this analysis is included as supplementary Table S3.

This method for calculating the probability of a gene cluster occurring by chance is vulnerable to statistical artifacts introduced by redundancy in annotation sources, so it is necessary to use an annotation source looking at gene-level and not exon-level information. Additionally, it is necessary to systematically curate any gene-level probeset redundancy out of the annotation source used. We used release 32 of the Affymetrix annotation for our array platform, which contains no exon-level information. Further, we chose the probesets with the lowest P-values to represent their genes, then deleted all other probesets representing each individual gene to get rid of any remaining gene-level redundancy. We manually inspected the data contributing to each significant finding to confirm that they were not redundant or otherwise problematic.

Shared synteny analysis utilized the homology map on the NCBI’s website, which has a map showing homologous genome regions between humans and mice. Graphics were generated using the positional information present in the homology map.

### Functional Network Analysis

We utilized a network analysis methodology similar to that used in a recent biological systems meta-analysis done on human BPD datasets [54]. Using a list of every gene with an FDR-adjusted P-value less than or equal to 0.25 from the gene-level microarray inferential statistics, we utilized the functional annotation clustering tool of NIAID’s DAVID software [100], [101]. We set the functional annotation tool to default settings with medium stringency and a background matching the array platform we used. The most significantly enriched cluster was related to ribosomes. Many of the genes in this cluster were pseudogenes, so we decided to ignore this cluster for the purposes of gene network analysis. The second most significant cluster we found had gene components generally related to chromatin packaging. We took the genes from this cluster and put them into the MiMI plugin v. 3.11 [102] in Cytoscape v. 2.8.0, setting the software to query genes with shared nearest neighbors in order to get rid of genes not closely related to the chromatin packaging network. The resulting network was large, with 183 nodes and 6234 edges. To reduce this network to the genes most closely related to chromatin packaging, we used the Glay plugin v. 2.0 [103] to find subnetworks. We found the subnetwork most closely related to chromatin remodeling, which is what we report in this study.

## Supporting Information

Table S1 Tab-delimited text file containing exhaustive microarray statistical analysis for all transcript clusters organized by P-value. Annotation sources from build 32 of the Affymetrix annotation for the Mouse Gene 1.0 ST platform with presumptive annotation filled in by Ensembl’s BioMart extension.
(TXT)

Table S2 Portable document file containing all information on RT-qPCR assays considered essential by the MIQE.
(PDF)

Table S3 Excel file containing results from the genome enrichment analysis broken down by chromosome.
(XLSX)
