# Supplementary material for: A New Mouse Model for Mania Shares Genetic Correlates with Human Bipolar Disorder
Source: PLoS One. 2012 Jun 4;7(6):e38128. doi: 10.1371/journal.pone.0038128 (PMC3366954; doi:10.1371/journal.pone.0038128)
Supplement: Table S2 — Portable document file containing all information on RT-qPCR assays considered essential by the MIQE. (PDF) [file pone.0038128.s002.pdf]

SUPPLEMENT S2: RT-QPCR METHODS

Table S2.1 – Experimental design

Definition of experimental and control groups	Experimental: Madison (MSN) mouse strain. Control: outbred hsd:ICR (ICR) mouse strain.
Number in each group	8 MSN, 8 ICR. All mice were male.
Assay carried out by core or investigator's laboratory	Carried out in investigator's laboratory.
Authors' contributions to qPCR section	C. Michael Saul: all molecular work, half of dissection work, writing.<br>Griffin M. Gessay: half of dissection work.<br>Stephen C. Gammie: bred mice, provided funding and lab space, writing.

Table S2.2 – Tissue Samples

Description	Fresh frozen whole hippocampus.
Volume or Mass of sample	See table S2.10.
Dissection Type	Gross dissection of hippocampal tissue from brain.
Processing Procedure	Animals were euthanized by cervical dislocation under isoflurane anesthetic, decapitated, and their hippocampi were immediately dissected from their brains.
If frozen, how quickly?	Samples frozen immediately on dry ice upon dissection.
Sample storage conditions	Stored at -80°C for no more than 12 weeks prior to RNA extraction.

Table S2.3 – Nucleic Acid Extraction

Procedure and/or instrumentation	Mortar and pestle disruption, guanidinium thiocyanate-phenol-chloroform extraction, and spin column cleanup and purification.
Name of kit and details of any modifications	Bio-Rad Aurum Total RNA Fatty and Fibrous Tissue Kit (catalog number 732-6830) used according to the manufacturer's specifications.
Sources of additional reagents used	Chloroform (Acros Organics, catalog number AC42355-0250)<br>Ethanol (Fisher Scientific, catalog number BP2818-500)
Details of DNase treatment	On-column treatment with DNase I according to manufacturer's specifications.
Contaminaion assessment of input RNA	NanoDrop curves used to assess presence of presence of protein, salt, and organic contaminants. All curves indicated clean samples.
Nucleic acid quantification	See table S2.10.
Instrument and method of nucleic acid quantification	NanoDrop spectrophotometer, absorbance at 260nm.
Purity (A_{260}/A_{280})	See table S2.10.
Yield	See table S2.10.
RNA integrity instrument	Agilent RNA 6000 Nano Chips with Agilent BioAnalyzer 2100.
RIN	See table S2.10.
Inhibition testing	C_q dilution, 1:8 using Ywhaz. As expected, diluted samples ran ~3 cycles behind undiluted samples.

Table S2.4 – Reverse Transcription

Complete reaction conditions	500µM dNTP mix, 20mM Tris-HCl (pH 8.4), 50mM KCl, 5mM $MgCl_2$, 2.5mM dT 20mers, 10mM DTT, 2U/µL RNaseOUT, 10U/µL SuperScript III RT.
Amount of RNA and reaction volume	25µL reactions, 2µg total RNA used in each reaction. 1.25µL RNAse H added after reaction termination.
Priming oligonuclotide and concentration	oligo-dT 20mers, final reaction concentration of 2.5mM.
Temperature and time	Prior to cDNA synthesis, RNA, primers, and dNTPs were denatured together at 65°C for 5 min. cDNA synthesis took place at 50°C for 50 min followed by an 85°C reaction termination step for 5 min. After reaction termination, the RNase reaction ran at 37°C for 20 min.
Manufacturer of reagents and catalog number	Invitrogen SuperScript III First-Strand Synthesis System for RT-PCR (catalog number 18080-051)
C_q with and without RT	See table S2.10 for no RT C_qs. While some gDNA contamination is present, its effect on experiments are negligible and stochastic according to hypothesis testing on no RT controls. This contamination is mostly a source of random error.
Storage conditions of cDNA	Stored at -80°C for no longer than 6 months.

Table S2.5 – qPCR Target Information

Gene symbol	See table S2.11.
Accession number	See table S2.11.
Location of amplicon	See table S2.11.
Amplicon length	See table S2.11.
In silico specificity	All primers screened for specificity using NCBI Primer-BLAST.
Homologs amplified	No primers amplified pseudogenes or retropseudogenes.
Sequence alignment	Aligned in NCBI Primer-BLAST.
Location of each primer by exon or intron	See table S2.11.
Targeted splice variants	Each primer set targets all splice variants for every transcript of interest as they are documented in the NCBI RefSeq RNA database.

Table S2.6 – qPCR oligonucleotides

Primer sequences	See table S2.11.
Probe sequences	Not applicable; dsDNA binding dye chemistry used.
Location and identity of any modifications	No modifications.
Manufacturer of oligonucleotides	UW-Madison Biotechnology Center DNA Synthesis Facility.
Purification method	Standard desalting and lyophilization.

Table S2.7 – qPCR protocol

Complete reaction conditions	2X Bio-Rad SsoFast EvaGreen Super Mix without ROX (catalog number 172-5204) used with no modifications.
Reaction volume and amount of cDNA/DNA	20µL reactions; 2µL 1:5 diluted cDNA used in each reaction.
Primer Concentration	500nM forward and 500nM reverse primer for all primer sets.
Mg^{2+} concentration	3.0mM $MgCl_2$.
dNTP concentration	200µM each of dATP, dTTP, dCTP, and dGTP. 800µM dNTP total.
Polymerase identity	Bio-Rad SsoFast Taq Fusion Polymerase.
Polymerase concentration	Proprietary concentration.
Buffer identity and manufacturer	Bio-Rad qPCR buffer provided with SsoFast EvaGreen Supermix.
Exact buffer chemistry	Proprietary composition.
PCR additives used	No additives used.
Manufacturer of plates and catalog number	Applied Biosystems MicroAmp Fast 96-Well Reaction Plates (catalog number 4346907).
Complete thermal cycling parameters	Incubation stage: 30s at 95°C. Cycling stage: 40 cycles, 3 steps: 5s at 95°C, 20s at annealing temperature (see table S2.11 for the specific annealing temperature used with each primer set), and 20s at 72°C.
Reaction setup	Manual using Eppendorf single channel adjustable volume pipettes.
qPCR instrument	Applied Biosystems StepOnePlus.

Table S2.8 – qPCR validation

Evidence of optimization	Prior to analysis, we ran each primer set at several annealing temperatures. We used the annealing temperature with the earliest C_q and the highest efficiency.
Specificity	Stringent *in silico* testing of primers prior to qPCR using Primer-BLAST, dissociation curve test of specificity *in vitro*.
C_q of NTC	C_q > 40 for all NTCs for all genes.
Calibration curves with slope and y-intercept (m, b)	See table S2.12.
Efficiency calculated from slope	See table S2.12.
r^2 of calibration curve	See table S2.12.
Linear dynamic range (LDR)	See table S2.12.
C_q variation at Limit of Detection	LOD measurements not necessary for relative quantification.
Evidence for LOD	LOD measurements not necessary for relative quantification.
If multiplex, efficiency and LOD for each assay	Not applicable; dsDNA binding dye chemistry used.

Table S2.9 – Data analysis

qPCR analysis program	Relative Expression Software Tool (REST)
Method of C_q determination	Used ABI StepOnePlus software to determine ABI's C_t value.
Outlier identification and disposition	Our experiments contain no outliers.
Results from NTC	All NTCs have no amplification.
Justification of number and choice of reference genes	The combination of Sdha and Ywhaz was found to be the most stable combination of reference genes by Gubern et al. (2009).
Description of normalization	Data normalized for baseline fluorescence.
Number and stage (reverse transcription or qPCR) of technical replicates	3 qPCR technical replicates.
Statistical methods for results significance	Randomization test for significance.
Software (source, version) of stats	StepOnePlus 2.1; REST 2009.

Table S2.10 – Sample quality control

ID	*Strain*	*Tissue Mass*	*RNA Concentration*	*RNA Yield*	*C_q (RT/no RT)*[†]	*A_{260} : A_{280}*	*RIN*
1	MSN	35.8 mg	222.34 ng/µL	35.574 µg	18.20/36.78*	2.11	8.2
2	MSN	36.7 mg	248.83 ng/µL	39.813 µg	17.84/40.00*	2.12	8.3
3	MSN	35.7 mg	271.41 ng/µL	43.426 µg	18.01/31.40	1.99	8.3
4	MSN	35.8 mg	235.50 ng/µL	37.680 µg	17.75/38.96*	2.12	8.2
5	MSN	60.0 mg	454.53 ng/µL	72.725 µg	18.09/36.62	2.08	8.4
6	MSN	48.2 mg	331.09 ng/µL	52.974 µg	17.88/35.23	2.11	8.2
7	MSN	41.0 mg	290.64 ng/µL	46.502 µg	17.83/38.10*	2.11	8.5
8	MSN	46.0 mg	263.10 ng/µL	42.096 µg	17.78/38.97*	2.11	8.3
9	ICR	50.7 mg	347.06 ng/µL	55.530 µg	18.03/34.77	2.09	8.5
10	ICR	32.2 mg	215.83 ng/µL	34.533 µg	17.66/36.45	2.12	8.2
11	ICR	44.2 mg	261.85 ng/µL	41.896 µg	17.77/33.17	2.13	8.3
12	ICR	38.2 mg	247.66 ng/µL	39.626 µg	17.83/39.20*	2.12	8.3
13	ICR	42.0 mg	278.96 ng/µL	44.634 µg	17.97/37.30*	2.11	8.9
14	ICR	37.2 mg	274.48 ng/µL	43.917 µg	17.98/38.91*	2.12	8.7
15	ICR	30.7 mg	236.85 ng/µL	37.896 µg	17.83/36.06	2.12	9.2
16	ICR	43.3 mg	288.02 ng/µL	46.083 µg	18.02/35.53	2.12	9.4

[†]RT versus no RT data were collected using the reference gene Ywhaz. C_q = 40 indicates no amplification detected.

**No amplification was detected in at least 1 of the replicates in these no RT controls.*

Table S2.11 – Primers

Gene Symbol	*RefSeq Accession*	*Primer Sequence (5'-3')*	*Product Length*	*Amplicon Location*	T_m
Ywhaz	NM_01174	F: TCCTTATTCCCTCTTGGCAG<br>R: ATGGAAGCTACATTAGCGGTTT	92 bp	Exon 5; 2432-2523 (3' UTR)	58°C
Sdha	NM_02328	F: CCGCTCCTACTGATGAAACC<br>R: GCGCAACTCAATCCCTTAC	179 bp	Exon 12; 2015-2193 (ORF, 3' UTR)	58°C
P2x7	NM_011027	F: CGAATTATGGCACCGTCAA<br>R: TCTCCGTCACCTCTGCTATG	150 bp	Exons 1, 2; 234-383 (ORF)	57°C
Epor	NM_010149	F: GTCCGATTCTGGCATCTCA<br>R: GGACAAGGCTGTTCTCATAG	107 bp	Exon 8; 1519-1625 (ORF)	58°C
Fhit	NM_010210	F: CAAACGATTCCCAAGGCATAA<br>R: GGGTACAATAAAGAGTGGTTAG	89 bp	Exon 7; 697-763 (3' UTR)	58°C
Cmklr1	NM_00815	F: ATCTTACACCATCATGCCACG<br>R: GTATACACACTGAAGCAAAGAGC	95 bp	Exon 3; 2003-2097 (3' UTR)	58°C
Npsr1	NM_175678	F: GTAGAGGAGCCAATTAACAAGTA<br>R: TAGACCAGAACTTGACAGAGAT	106 bp	Exon 10; 2866-2971 (3' UTR)	57°C
Tac1	NM_009311	F: ACGCACTATCTATTCATCTTCATC<br>R: AGAATTACAAGGCTTATTGGCA	167 bp	Exon 7; 502-668 (3' UTR)	58°C
Cat	NM_009804	F: TTCCCACTTGGATTATGTTGATG<br>R: CTGAAAGCAACCAAACACGG	119 bp	Exon 13; 2358-2476 (3' UTR)	56°C

Table S2.12 – qPCR quality control

Gene Symbol	*PCR Efficiency*	*Linear Dynamic Range*	r^2	*Slope*	*y Intercept*
Ywhaz	98.375%	C_q: 17.60-27.71	0.998	-3.362	25.020
Sdha	97.271%	C_q: 17.97-28.19	0.998	-3.389	25.472
P2x7	96.354%	C_q: 27.06-33.29	0.989	-3.412	34.583
Epor	99.573%	C_q: 25.42-31.47	0.998	-3.332	32.743
Fhit	104.902%	C_q: 26.25-33.86	0.990	-3.210	33.346
Cmklr1	98.375%	C_q: 27.92-35.92	0.994	-3.362	35.382
Npsr1	99.305%	C_q: 30.68-34.69	0.989	-3.339	38.136
Tac1	97.414%	C_q: 23.69-29.79	0.997	-3.385	31.181
Cat	97.228%	C_q: 28.24-34.31	0.987	-3.390	35.793
